# Antimicrobial Polymeric Composites with Embedded Nanotextured Magnesium Oxide

**DOI:** 10.3390/polym13132183

**Published:** 2021-06-30

**Authors:** Nemanja Aničić, Mario Kurtjak, Samo Jeverica, Danilo Suvorov, Marija Vukomanović

**Affiliations:** 1 Advanced Materials Department, Jozef Stefan Institute, Jamova 39, 1000 Ljubljana, Slovenia; nemanja.anicic@gmail.com (N.A.); mario.kurtjak@ijs.si (M.K.); danilo.suvorov@ijs.si (D.S.); 2 National Laboratory of Health, Environment and Food, Prvomajska 1, 2000 Maribor, Slovenia; samo.jeverica@gmail.com

**Keywords:** magnesium oxide, nanotextured surface, antimicrobial, polymer-matrix composites, drug release, contact-based antimicrobial surface

## Abstract

Nanotextured magnesium oxide (MgO) can exhibit both antibacterial and tissue regeneration activity, which makes it very useful for implant protection. To successfully combine these two properties, MgO needs to be processed within an appropriate carrier system that can keep MgO surface available for interactions with cells, slow down the conversion of MgO to the less active hydroxide and control MgO solubility. Here we present new composites with nanotextured MgO microrods embedded in different biodegradable polymer matrixes: poly-lactide-co-glycolide (PLGA), poly-lactide (PLA) and polycaprolactone (PCL). Relative to their hydrophilicity, polarity and degradability, the matrices were able to affect and control the structural and functional properties of the resulting composites in different manners. We found PLGA matrix the most effective in performing this task. The application of the nanotextured 1D morphology and the appropriate balancing of MgO/PLGA interphase interactions with optimal polymer degradation kinetics resulted in superior bactericidal activity of the composites against either planktonic *E. coli* or sessile *S. epidermidis*, *S. aureus* (multidrug resistant-MRSA) and three clinical strains isolated from implant-associated infections (*S. aureus*, *E. coli* and *P. aeruginosa*), while ensuring controllable release of magnesium ions and showing no harmful effects on red blood cells.

## 1. Introduction

Magnesium exhibits an essential role in bone mineralization, and its deficiency is a risk factor for osteoporosis [1,2]. It promotes proliferation and differentiation of osteoblasts and inhibits osteoclastogenesis [3]. Similarly to Ca^2+^, Mg^2+^ ions activate integrins for ligand binding and positively influence attachment and migration of osteoblasts [4,5]. For these reasons Mg^2+^ ions have been incorporated into various ceramic-based platforms for orthopaedic biomaterials to improve their bioactivity and body integration [6,7].

Magnesium oxide (MgO) is a particularly interesting source of Mg^2+^, highly applicable for designing bioactive surfaces in medical devices. Impregnation of MgO with poly(L)lactic acid (PLA) results in composites with very promising synergistic effects [4,8]: while PLA establishes stabilization of the MgO nanoparticles (NPs) and the delivery of the Mg^2+^ ions, MgO enhances the mechanical properties of the PLA and reduces the toxic effect of the acidic PLA degradation products [4,8]. As a consequence, addition of MgO NPs to PLA improves the viability and the attachment of the osteoblasts [4]. At the same time, it reduces the inflammatory potential of the acidic products of hydrolytic PLA degradation [8]. Apart from being a source of bioactive Mg^2+^ ions, another useful property of nanosized MgO is its robust antibacterial activity [9]. Several studies have highlighted the contact-based bactericidal potential of MgO NPs against a broad range of bacteria [9,10,11,12]. Our recent investigations shed light on the mechanism of this contact-based antibacterial action and revealed that MgO microrods (MRs) can also possess antibacterial properties if they are carefully designed by nanoengineering the texture of their surfaces [9,12]. Nanotextured MgO MRs exhibit even stronger and more efficient bactericidal action than MgO nanoparticles with similar crystallite size despite a notably lower specific surface area. The improved activity is attributed to better contact with bacteria and better resistance to hydrolysis in the case of nanotextured MgO MRs [9,12].

Coupling antimicrobial and bioactive properties within MgO NPs impregnated with PLA has been presented as an interesting strategy to protect implant’s surface [13]. Preventing bacterial colonisation and biofilm formation while ensuring optimal tissue integration [14,15] is particularly useful during biomaterial implantation and can prevent serious medical complications [16,17]. MgO NPs stimulate the differentiation of bone marrow stem cells into osteoblasts and disturb the attachment of bacteria to the MgO surface without affecting bacterial viability (at 0.2 g/L concentration) [13]. However, at 0.3 g/L they already started to exhibit cytotoxicity while still not affecting the viability of the bacteria in the solution [13]. Such findings demonstrate a narrow therapeutic window for application of MgO NPs, which pointed out a necessity to sensibly design an appropriate system for controlled delivery of MgO to be able to exploit both, its antibacterial and bioactive properties. Degradable polyesters (e.g., poly (lactic-co-glycolic) acid (PLGA), PLA and polycaprolactone (PCL)) are already established as effective, FDA approved drug-delivery systems [18,19] and could be optimal options for designing a balance between bioactivity and antimicrobial activity of composites containing MgO.

Hence, the aim of this work was to design effective antimicrobial composites composed of nanotextured MgO in polymeric matrix by exploring the correlation between their structure and antibacterial function against clinically relevant and antibiotic-resistant bacterial strains.

## 2. Materials and Methods

### 2.1. Synthesis and Processing

MgO MRs were created by precipitation of a carbonate-based 1D precursor and subsequent 3 h calcination at 900 °C for 3 h in room air (according to [9,12]). A solvent-casting method was used to prepare the composite coatings. Commercial polymers were used: PLGA (monomer ratio 50:50, Resomer^®^ RG 505, Evonik Industrie, Essen, Germany), PLA (Resomer^®^ LR 704 S, Evonik industrie, Essen, Germany) or PCL (99 % purity, Mw = 100,000, Sigma Aldrich, St. Louis, MO, USA). Polymeric (PLGA, PLA or PCL) solution in acetonitrile (40 g/L) was mixed with a suspension of MgO in acetonitrile and left in either a 20 mL vial or in a microtiter plate (MTP) under the hood overnight to allow for the solvent evaporation. The final mass per surface area of the composite coatings was 7.5 mg/cm^2^. The fraction of the embedded MgO was varied from 1 wt% to 25 wt% (mass of MgO per mass of composite).

### 2.2. Composite Characterization

The morphology of the MgO/polymer composites was examined by scanning electron microscopy (SEM, Jeol JSM-7600F, JEOL, Tokyo, Japan) using secondary electrons for imaging and operating at 1.5–5 kV. The composites were stuck on a carbon tape and coated with carbon before observation. FT-IR analyses were conducted on a Perkin Elmer Spectrum 400 MIR spectrophotometer (Perkin Elmer, Waltham, MA, USA) in the attenuated total-reflection (ATR) technique, with 10 scans per sample and a resolution of 4 cm^−1^.

### 2.3. Magnesium Release from MgO/Polymer Composites

A total of 20 mg of composite were exposed to 6 mL of physiological solution (0.9% NaCl) at 37 °C and linear shaking at a rate of 60 rpm for 6 days. Then, 500-µL aliquots were taken out (and replaced with fresh medium) every 24 h for analysis of Mg content. The experiment was performed in quadruplicates. A cumulative amount of released magnesium was plotted, so that all the magnesium in the previous aliquots was added to the measured amount after each day.

### 2.4. Quantification of Released Magnesium

The released Mg was determined using 4-(2-pyridylazo)-resorcinol (PAR) and 0.5 M TRIS buffer solution in deionized water [20]. A total of 300 µL of the reagent solution (0.054 mg of PAR in 50 mL of 0.02 M KOH) was mixed with 200 µL of 0.5 M TRIS buffer and 100 µL of sample. Deionized water was added up to a total volume of 1 mL. Absorbance was measured at 506 nm in a multiplate reader (Synergy H1, Biotek, Winooski, VT, USA). Standard solutions for a calibration curve were prepared in the concentration range from 0.025 mg/L to 4 mg/L using MgCl_2_ 6H_2_O. When the concentration in a sample was out of the linear range, it was further diluted with water before adding PAR and TRIS buffer.

### 2.5. Characterisation of Composites after Exposure to Physiological Solution

The three composites (MgO/PLGA, MgO/PLA and MgO/PCL) were characterised by SEM and ATR IR spectroscopy after their exposure to 0.9% NaCl (composites containing 25% of MgO for 2 h, 4 h and 24 h, whereas MgO/PLGA0.10 and MgO/PLA0.10 for 8 h and 6 days). The composites were carefully removed from the vials, dried without washing and observed under SEM and their IR spectra were measured.

### 2.6. Metabolic Activity of Attached Bacteria

The metabolic activity of bacteria was measured using resazurin fluorescence assay [21] for *S. epidermidis* (NCIMB 8853), *S. aureus* (ATCC 43300-MRSA) and three clinical strains isolated from implant-associated infections, *S. aureus* (16/4856), *E. coli* (16/7613) and *P. aeruginosa* (14/8011), which colonized either the PLGA (negative reference) or MgO/polymer coatings’ surfaces. A blank PLGA sample (without bacteria, incubated in sterile medium) was used to check the sterility of the conditions. The surface density of the coating was 7.5 mg/cm^2^ within MTPs, while the fraction of MgO in the composite was varied as 1, 5, 10 or 25 wt%. The overnight liquid culture of bacteria in thioglycolate broth was centrifuged to remove excess medium and dissolved in physiological solution supplemented with 1% glucose (*w*/*w*) to enhance biofilm attachment on the coating. Bacterial suspension of 0.5 McFarland standard turbidity was prepared, and 200 µL were transferred to MTPs for a 24-h incubation at 37 °C. Measurements were performed in eight replicates. A total of 250 µL of resazurin (20 mg/L, physiological solution) was added to each well and incubated for 2 h at 37 °C. Aliquots were transferred to a 96-well MTP for fluorescence measurements at 530/590 nm.

### 2.7. Viability of Attached Bacteria

Live/dead assay (L70007 *Bac*Light, Molecular Probes) was used to detect bacterial viability by fluorescence microscopy. The bacterial colonization of the coatings (either PLGA or MgO/PLGA) was done as in Section 2.6, but in 20 mL glass vials instead of MTPs. The coatings were rinsed in physiological solution, stained with live/dead kit solution for 15 min and observed in Nikon Eclipse Ti-U inverted microscope (Nikon, Tokyo, Japan).

### 2.8. Lethal Potential for Planktonic Bacteria

The live/dead assay was also utilized to monitor the lethal potential of MgO/PLGA against *E. coli* (ATCC 47076, Eugene, OR, USA). Coatings with 7.5 mg/cm^2^ surface density and 10 wt% MgO content were immersed in 2 mL of bacterial suspension. At regular time intervals, 100 µL aliquots were taken out and stained with live/dead kit solution for 15 min. The fraction of the live bacteria was determined from the green-to-red fluorescence ratios [22]. Each measurement was performed in 4 replicates.

### 2.9. Haemolysis Assay

Fresh sterile whole sheep blood was supplied by the Institute of Microbiology and Parasitology (Veterinary Faculty, University of Ljubljana, Ljubljana, Slovenia). The withdrawal of the blood was performed with sterile accessories from healthy animals and was in accordance with the national guidelines (No. 510-05/13-3/2). Blood was stored in the Alsever’s medium and used within 7 days as a source of red blood cells (RBCs). Alsever’s medium contained 4.2 g/L of NaCl (Carlo Erba Chemicals, Val de Reuil, France, 99%), 2.17 g/L of sodium citrate dihydrate (Sigma Aldrich, 99.8%) and 20 g/L of D-glucose (Sigma Aldrich, 99.5%). The experiments on RBCs were performed in accordance with the national regulations (No. 003-02-5/2011-26) and biosafety guidelines implemented at the Jožef Stefan Institute. RBCs were diluted in HEPES (Sigma Aldrich, 99.5%) to a final concentration of 5 × 10^7^ RBC/mL. They were incubated with the coatings (7.5 mg/cm^2^) or MgO particles (1 mg/mL) for 24 h at 37 °C. PLGA and blank sample (without any material) were used as negative references. After the exposure, the cells were centrifuged at 1000 g for 10 min and supernatants were evaluated for pH value (Eutech PC700, Thermo Fisher Scientific, MA, USA) and haemoglobin content (by measuring UV/V is absorption at 415 nm [23]). The measurements were performed in four replicates.

### 2.10. Morphology of RBCs or Bacteria on Coatings

The coatings incubated with RBCs or bacteria were gently washed in physiological solution and incubated for 30 min in 2% glutaraldehyde solution (Sigma Aldrich). Each coating was then frozen in liquid N_2_ and lyophilized. Before examination in SEM (using low-energy electrons and 2–5 kV voltage), the lyophilized coatings were placed on a sticky carbon tape and coated with 5 nm of platinum in a Precision Etching Coating System (PECS Model 682, Gatan, Pleasanton, CA, USA).

### 2.11. Statistical Analysis

Each sample population (at least four replicates) was tested for the existence of outliers. We used a two-sided *t*-test for independent samples to assess the statistical significance of the differences between the two sample averages (Origin 2015, OriginLab, Northampton, MA, USA).

## 3. Results

### 3.1. Structural Properties of MgO/Polymer Composites

**Figure 1:**
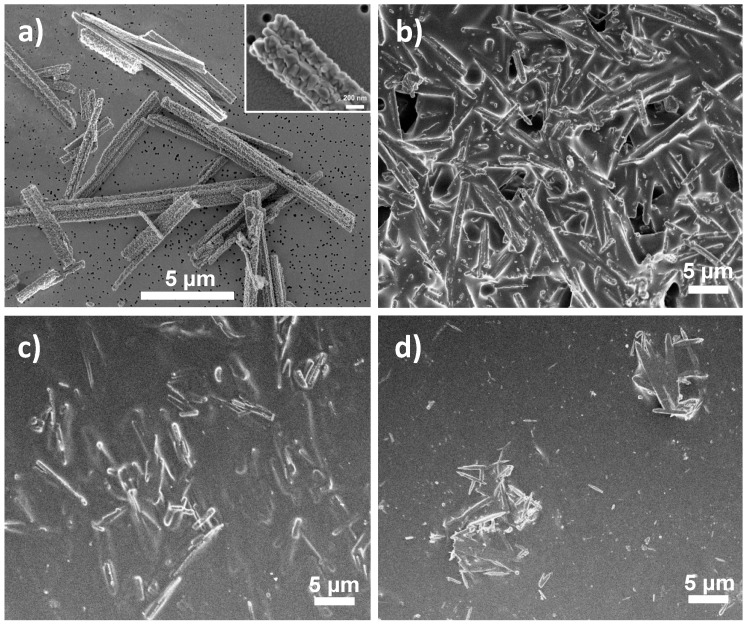
Morphology of (**a**) nanotextured MgO MRs and different MgO/polymer composites with 25 wt% MgO content under SEM: (**b**) MgO/PLGA, (**c**) MgO/PLA, (**d**) MgO/PCL.

MgO/polymer composites containing three different polymeric matrices (PLGA, PLA and PCL) were prepared as films with constant mass per surface area (7.5 mg/cm^2^) and different weight fractions of MgO (1, 5, 10 or 25 wt%, expressed as initial mass of MgO per mass of composite). Most of the presented characterisation was done on composites with 25 wt% of MgO, for which the differences between the composites were more obvious. The surface morphologies of the MgO/polymer composites and MgO without polymer are compared in Figure 1. The microrod shape of the MgO component can be distinctively observed in all three composites and the nanotexturing of the surfaces of MgO MRs (Figure 1a) is not visible in any case (Figure 1b–d). The MRs lie underneath the polymer surface, almost aligned with the surface plane and completely covered by the polymer. The distribution of the MRs within the polymer matrix is different for different polymers. Laterally, MgO MRs appear to be more evenly and more densely distributed over the surface of PLGA (Figure 1b) than PLA (Figure 1c) and even less uniformly in the case of PCL, where separate agglomerates of MRs can be observed (Figure 1d). Mesially, the surface appears more populated with MgO in the case of PLGA (Figure 1b), whereas MgO MRs seem to lie deeper underneath in the case of PLA and PCL (Figure 1c,d).

**Figure 2:**
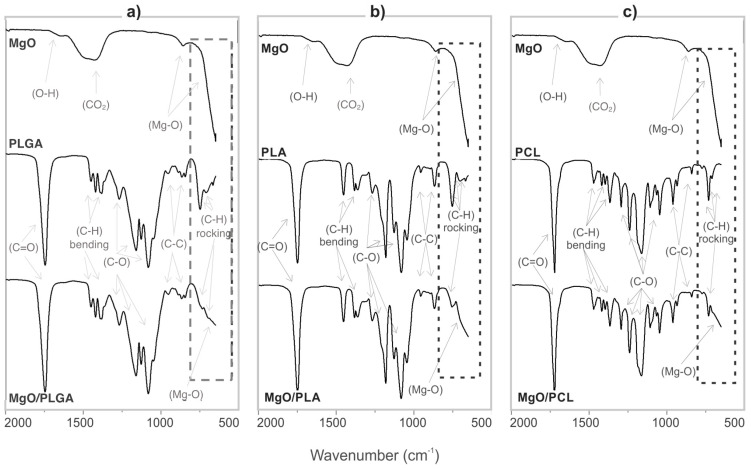
ATR-IR spectrum of (**a**) MgO, PLGA and MgO/PLGA; (**b**) MgO, PLA and MgO/PLA; and (**c**) MgO, PCL and MgO/PCL. The MgO mass fraction in the composite coatings was 25% and 0% in the reference polymer coatings, which were prepared in the same way as the composites.

The differences in the morphology pointed towards differences in the interphase interactions within the MgO/polymer composites, which were further investigated using IR spectroscopy. The ATR-IR spectrum of the nanotextured MgO (Figure 2) contains two Mg–O vibrations [24] (one at 860 cm^−1^ and another, only partially observed, below 650 cm^−1^) and IR absorption bands related to CO_2_ (at 1430 cm^−1^) and H_2_O (at 1650 cm^−1^) on the surface [12,25]. The ATR IR absorption bands of MgO are weak when compared to the polymer ones and even weaker inside the composites. The spectra of pure PLGA, PLA and PCL (Figure 2) exhibit characteristic poly-ester absorption bands [26,27,28], while the spectrum of MgO/PLGA consists of both Mg–O and PLGA vibrations (Figure 2a). The intensity of the C–H rocking vibrations is reduced for the composite in comparison with pure PLGA. The maximum intensity ratio between the C–H vibration band at 751 cm^−1^ and the C=O vibration band at 1747 cm^−1^ is 80% lower for MgO/PLGA than for pure PLGA (Figure 2a). A similar decrease of this ratio is observed for MgO/PLA and MgO/PCL as well. For MgO/PLA the C=O vibration band reduction is 70%, while in the case of MgO/PCL it is 55% (Figure 2b,c). This indicates that all three investigated polyesters predominantly interact with the MgO MRs via their C–H bonds and the interphase interactions weaken in the order: MgO/PLGA > MgO/PLA > MgO/PCL.

### 3.2. Release of MgO MRs from the MgO/Polymer Composites under In Vitro Physiological Conditions

To investigate the role of the polymer type (PLGA, PLA, PCL), the morphology and the interphase interactions on the composite stability and functionality, we examined the elution of magnesium and the composite properties after immersion in physiological solution.

**Figure 3:**
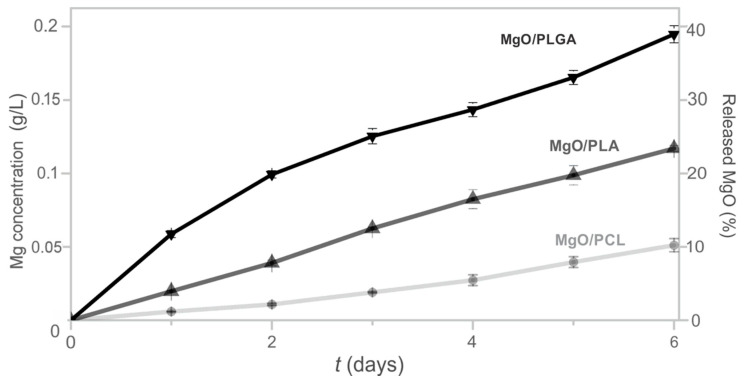
The impact of polymer matrix composition on the magnesium elution from MgO/polymer composites in 0.9% NaCl solution. Cumulative magnesium elution kinetics is presented as either an increase in magnesium concentration or percentage of released MgO from the polymer matrix. All three composites initially contained 25 wt% of MgO.

The magnesium release from the MgO/polymer composites was quantified by a method that does not discriminate, whether MgO was only released, dissolved or transformed to magnesium hydroxide in the physiological solution [29]. Thus, the total released Mg in the liquid above the coating was determined (Figure 3). This value was also converted to the fraction of MgO that was released from the composite. During the first day, 12% of MgO particles were released to the medium from the MgO/PLGA composite (Figure 3). Afterwards, the daily elution kinetics was stabilized in the range of 4–8% per day and after six days it reached 38% of MgO (Figure 3). The magnesium release kinetics from the MgO/PLA and MgO/PCL composite coatings followed similar trends, but the extent of Mg elution was lower for the PLA polymer and even lower for PCL. After 6 days of exposure, 23% of MgO was released from MgO/PLA and 10% from the MgO/PCL composites. One may observe that the release trend follows the polarity of the polymer matrices and the surface density of the MRs in the composites. The most polar matrix (PLGA) with the highest population of MgO MRs closer to the surface (according to the SEM analysis in Figure 1) exhibits the highest release of the nano-textured MgO MRs.

**Figure 4:**
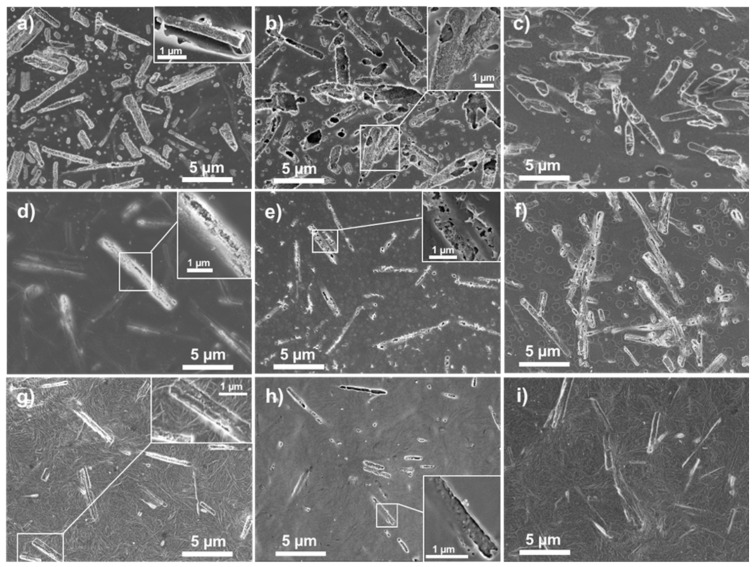
SEM visualised surface morphology of: MgO/PLGA composite coating after exposure to physiological solution and linear shaking for (**a**) 2 h, (**b**) 4 h and (**c**) 24 h; MgO/PLA composite coating after exposure to physiological solution and linear shaking for (**d**) 2 h, (**e**) 4 h and (**f**) 24 h; MgO/PCL composite coating after exposure to physiological solution and linear shaking for (**g**) 2 h, (**h**) 4 h and (**i**) 24 h.

Immersion of the MgO/polymer composites into the physiological solution for two hours already caused notable changes in their morphology. The surface of the MgO/PLGA coating contained smaller spherical holes (up to 300 nm in size) due to PLGA degradation and larger holes with 1D (gutter-like) shape corresponding to the shape of the MgO MRs. Within most of them, the MgO MRs were situated after 2 h (Figure 4a). In contrast to the coating before exposure (Figure 1b), after 2 h of immersion the MgO MRs became partially exposed, as their nano-texturing was once again established (Figure 4a, enlarged area). Thus, PLGA matrix was able to retain the nano-textured MgO MRs on its surface. After 4 h, the fraction of empty rectangular holes increased, but there were still many holes that contained MgO MRs with a fully exposed nanotextured surface (Figure 4b). After 24 h, we can see rectangular holes with additional holes inside uncovering new MgO MRs deeper inside, apart from rectangular holes filled with MgO particles (Figure 4c). It seems that after the upper layer of the first 1D holes had been emptied, the degradation continued deeper inside and a second layer of exposed MgO started emerging. On the other hand, the MgO particles that were still coated by PLGA after 2 or 4 h, have become exposed later.

As PLA and PCL degrade slower than PLGA in physiological conditions [30], the surfaces of MgO/PLA and MgO/PCL were free from small spherical holes after 2 h (Figure 4d,g). Moreover, the majority of MgO MRs was still situated under the surface and fully covered with polymer (their nano-textured surfaces were not visible). Only few 1D-shaped holes could be observed, and they were narrower than in MgO/PLGA, they looked more like cracks (Figure 4d,g). The nanotextured surface of the MgO lying underneath was partially revealed in some of them (inset of Figure 4d). This indicates that within the first 2 h of incubation, when no significant amount of magnesium had been released yet, the nanotextured surface of MgO was already available at the surface of MgO/PLGA, but not in MgO/PLA or MgO/PCL. After 4 h, cracks appeared above most of the MgO close to the surface of MgO/PLA, but they were still not wide enough to fully expose their nanotextured surface (Figure 4e). Only after 24 h the surface of MgO/PLA looked similar to the MgO/PLGA surface after 2 h, and even then, the proportion of fully exposed MRs was smaller in the MgO/PLA composite (Figure 4f). A larger number and width of cracks was detected also in MgO/PCL after 4 h (Figure 4h). However, they were either empty or the uncovered nanotextured surface of MgO underneath was partially re-sealed by a thin layer of PCL (insets in Figure 4g,h). Consequently, most of the MRs were still covered by PCL even after 24 h in physiological solution (Figure 4i).

ATR IR spectroscopy revealed no general structural change after 2 h incubation in physiological medium apart from a noticeable decrease in the intensities of absorption bands due to degradation of PLGA. However, in case of MgO/PLA and, even more pronounced, in case of MgO/PCL, an additional peak at around 3700 cm^−1^ was observed (Figure S1a). The peak is related to O–H vibration of Mg(OH)_2_ phase due to partial transformation of MgO [12]. This was confirmed with the IR spectrum of pure MgO nanotextured MRs after 2 h incubation, in which the hydroxide absorption band was very intense (Figure S1a). There was no such vibration band in the IR spectrum of MgO/PLGA after 2 h. However, some hydroxide phase was present in all three composites after 24 h in physiological solution (Figure S1b). The signal was very low in case of MgO/PLGA, though.

MgO/PLGA and MgO/PLA composites with lower MgO content (10%) have also been investigated for their in vitro hydrolysis in physiological solution by IR spectroscopy (Figure S2). Mg(OH)_2_ could not be detected in case of MgO/PLGA0.10 neither after 8 h nor after 6 days. By contrast, there was a distinct hydroxide absorption band in MgO/PLA0.10 after 8 h, but it disappeared after 6 days.

In sum, these results emphasize the ability of the PLGA to prevent the phase transformation of the MgO MRs inside the composite within the first 2 h of exposure despite their greater exposure to the physiological medium due to the faster degradation of the polymer.

### 3.3. Antibacterial Properties of the MgO/Polymer Composite Coatings

**Figure 5:**
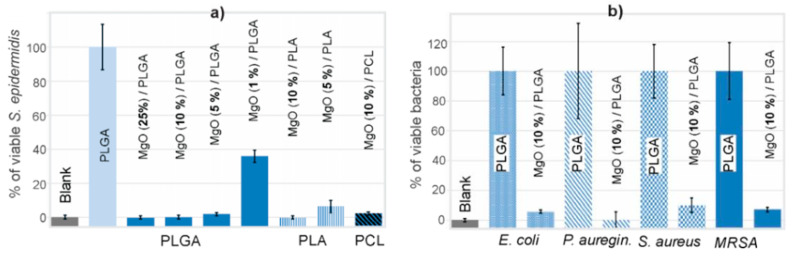
Relative metabolic activity of colonizing (**a**) *S. epidermidis* on the prepared MgO/polymer coatings and (**b**) *E. coli*, *P. aeruginosa*, *S. aureus* and MRSA on the MgO/PLGA coating. Blank sample is PLGA in sterile medium without bacteria.

Antimicrobial properties were initially evaluated by *S. epidermidis* surface colonization. The efficacy of the composites depended on the MgO content. The MgO/PLGA0.25 and MgO/PLGA0.10 composite coatings were completely effective against *S. epidermidis* colonization, as no metabolic activity was measured after the incubation (Figure 5a), while the MgO/PLGA0.05 and MgO/PLGA0.01 coatings were unable to suppress all the metabolic activity of the attached *S. epidermidis*, which retained 3% and 39% of the activity relative to the negative control (bacteria colonizing pure PLGA coating, Figure 5a), respectively. The MgO/PLA0.10 coating was completely effective against *S. epidermidis* colonization, whereas the MgO/PLA0.05 coating allowed *S. epidermidis* colonization with the resulting metabolic activity equal to 6%, relative to the negative control (Figure 5a). Hence, MgO/PLA coatings were less efficient than MgO/PLGA. The efficacy of MgO/PCL coating was even lower, as the PCL-based coating containing 10 wt% of MgO was not able to prevent colonization of *S. epidermidis* (3% activity relative to the negative control compared to 0% with MgO/PLA0.10, Figure 5a). Similar to the observations in the release study, the decrease in the antibacterial activity of the MgO/polymer composites (PLGA > PLA > PCL) follows the trend in the polarity of the polymers.

As the most effective coating, MgO/PLGA0.10 was further investigated by its ability to prevent colonization of several other clinically significant bacterial isolates from implant-associated infections. Measurements of the metabolic activity of the colonizing bacteria showed that the MgO/PLGA0.10 coating was able to reduce surface colonization of *E. coli* for 94%, *P. aeruginosa* for 100%, *S. aureus* for 90% and MRSA for 93%, in relation to the negative controls (Figure 5b). Thus, broad-range bactericidal activity of the MgO/PLGA coating against several clinically relevant and antibiotic resistant bacteria was also confirmed.

**Figure 6:**
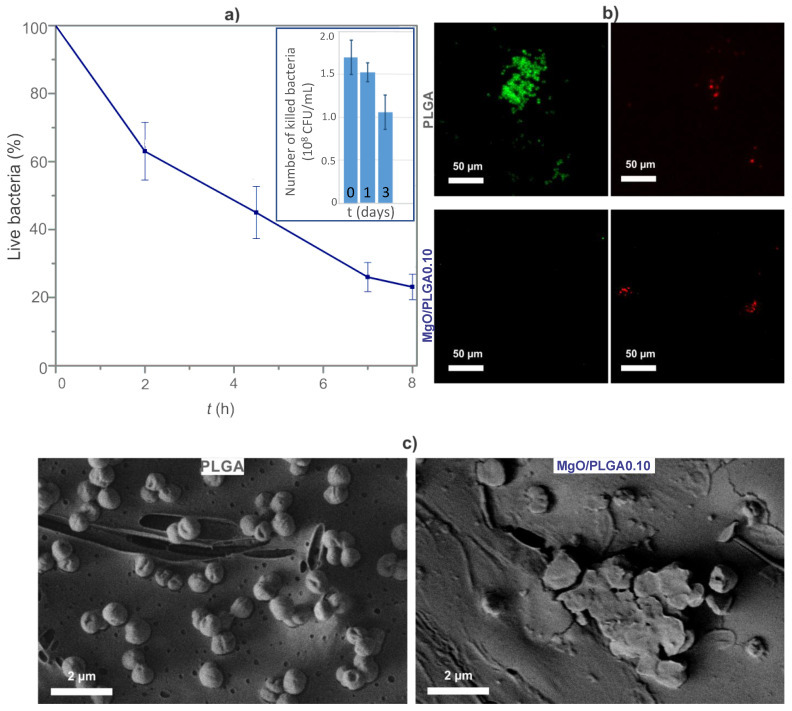
(**a**) The decrease in planktonic *E. coli* viability upon exposure to the MgO/PLGA0.10 composite coating; the decrease in bactericidal potential of the MgO/PLGA0.10 composite coating due to preceding exposure to physiological solution for either one or three days (chart insert); (**b**) survivability of *S. epidermidis* bacteria colonizing PLGA and MgO/PLGA0.10 coatings by BacLight live/dead fluorescent imaging; (**c**) SEM imaging of bacteria colonizing PLGA and MgO/PLGA0.10 coatings.

To determine the correlation between the magnesium release from the prepared MgO/PLGA composites and their prolonged bactericidal activity, the lethal kinetics of the MgO/PLGA0.10 coating before and after partial in vitro degradation was measured. The initial MgO/PLGA0.10 coating eliminated *E. coli* in a steady manner (Figure 6a). It eliminated 75% of all bacteria within 8 h of exposure. Then, this composite coating was subjected to physiological solution for one or three days and incubated with bacteria after washing. The lethal efficiency of MgO/PLGA0.10 coating was decreasing with an increasing length of pre-treatment. It decreased from 85% (fresh MgO/PLGA0.10 coating) to 75% (one-day old MgO/PLGA0.10 coating) and 53% (three-day old MgO/PLGA0.10 coating). Thus, over three days of the exposure the lethal potential dropped for 32% (Figure 6a).

The viability of the bacteria on the coating was also monitored by a live/dead fluorescence assay. *S. epidermidis* bacteria on the MgO/PLGA0.10 composite were compared with the ones on the PLGA without MgO. The clusters of bacteria on the PLGA surface emitted strong green signal indicating live bacteria (Figure 6b). By contrast, intensive red signal was emitted from the surface of the MgO/PLGA coating, indicating dead bacteria and the abundance of the *S. epidermidis* clusters on the surface of MgO/PLGA coating was much lower (Figure 6b). 

Furthermore, the morphological changes of *S. epidermidis* after the interaction with the MgO/PLGA0.10 coatings were also observed under SEM (Figure 6c). *S. epidermidis* on the surface of PLGA (strong metabolic activity, viability confirmed by the live/dead assay) exhibited typical undamaged spherical morphology. By contrast, the bacteria on the surface of MgO/PLGA0.10 (no metabolic activity, non-viability confirmed by the live/dead assay) appeared flattened with serious membrane damages. All three employed methods proved strong bactericidal potential and efficacy in prevention of bacterial colonization of the MgO/PLGA0.10 composite coating.

### 3.4. The Impact of the MgO/PLGA Composites on Red Blood Cells

**Figure 7:**
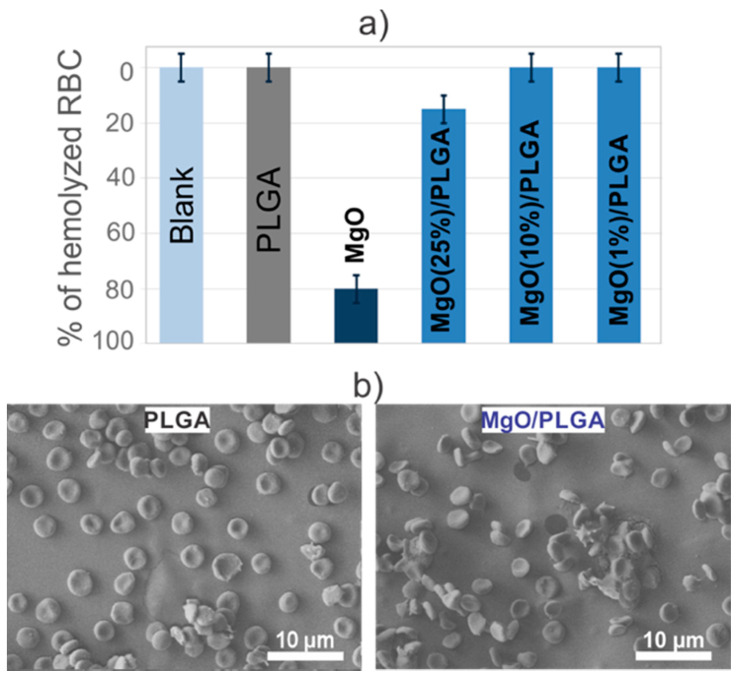
(**a**) Fraction of hemolysed red blood cells upon exposure to either MgO nanotextured MRs or MgO/PLGA coatings with different percentage of MgO content. Blank sample consists of only RBCs without any material. (**b**) Morphology of attached red blood cells on the PLGA and MgO/PLGA 0.10 coatings.

Since they exhibited the strongest antibacterial properties, the MgO/PLGA composite coatings were chosen for the hemocompatibility assay. After a 24-h incubation of the coatings with blood, the amount of released haemoglobin was measured. Using a previously prepared calibration curve (Figure S3), the proportion of haemolysed RBCs was determined. The results indicated that the exposure of the PLGA coating, as well as MgO/PLGA0.10 and MgO/PLGA0.01 coatings, to the blood solution did not cause haemolysis of RBCs. By contrast, 15% of RBCs were haemolysed upon contact with the MgO/PLGA0.25 coating (Figure 7a), while 80% of RBCs were haemolysed when exposed to a suspension (1 g/L) of nanotextured MgO MRs for 24 h. The change of pH during the incubation was also measured. The supernatant pH was reduced from 7.4 (HEPES) to 7.25 after the 24-h incubation of the PLGA coating in the HEPES blood suspension, but it remained at 7.4 in the case of MgO/PLGA0.10 coating, i.e., MgO scavenged acidic products of the PLGA degradation.

The determination of the extent of hemolysis provided information about the average impact of the prepared coatings on the blood dilution in the HEPES buffer. However, it is desirable to evaluate the impact of a coating on the RBCs in the vicinity of its surface. Therefore, after their exposure to either the PLGA or the MgO/PLGA0.10 coating, the morphology of the RBCs at the surface of the coating was observed by SEM. The RBCs at the surface of the PLGA coating exhibited slightly crenated shape instead of a perfect disc one, while the introduction of 10% of MgO within the PLGA matrix resulted in the reduction of the number of RBCs with crenated shapes attached to the coating surface (Figure 7b).

## 4. Discussion

**Figure 8:**
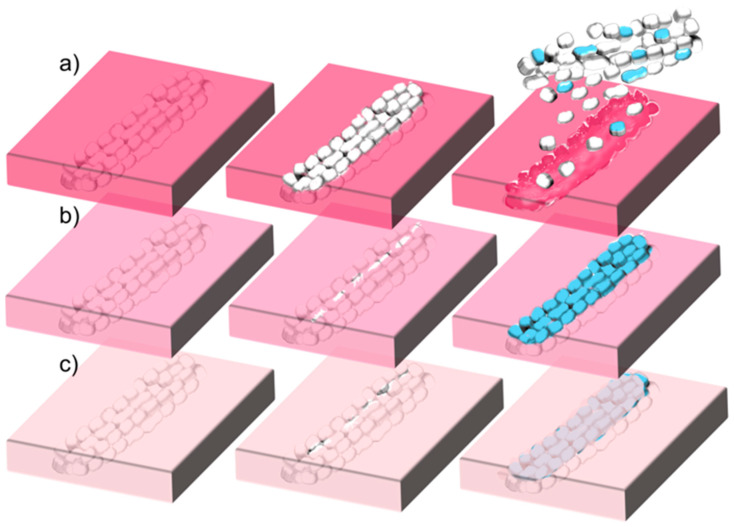
Schematic representation of the differences in the degradation behaviour that lead to different antibacterial efficiencies of the different MgO/polymer composites: (**a**) MgO/PLGA, (**b**) MgO/PLA and (**c**) MgO/PCL. Three stages are shown from left to right: before exposure to physiological solution, after short exposure (2–4 h) and after 1 day exposure. White nanotextured MR is composed of the active MgO, whereas the blue colour represents the less active hydroxide phase.

Our characterisation of three different MgO/polymer composites, prepared by a solvent casting method, and comparison of their in vitro bioeffective properties has shown that PLGA polymer, which enabled the fastest and highest release of MgO, also enabled the strongest antibacterial action of the composite. However, this result cannot be explained solely by the larger amount of released antibacterial component, as the concentration of released MgO is not high enough to kill planktonic bacteria (5–10 times lower than minimal bactericidal concentration [9,12]) to such extent that the biofilm formation on the coating would be so effectively reduced. Even lower concentration is expected for MgO/PLGA0.10, which exhibited similar antibacterial properties against *S. epidermidis* as MgO/PLGA0.25. Moreover, the differences in the released MgO are not large enough to account for the large differences in the antibacterial performance between the three composites. Therefore, the surface of the MgO/PLGA must be more effective against bacteria than the surfaces of the MgO/PLA and MgO/PCL. There are several reasons for this:

**(i) Different rates of polymer degradation.** This seems to be the most important contribution. Degradation of PLGA in the composite occurs evidently faster than degradation of PLA or PCL. Furthermore, faster degradation causes earlier exposure of the nanotextured antibacterial MgO MRs on the surface of the coating. Thus, the surface of the coating obtains antibacterial protection earlier. Due to the contact-based mechanism of the MgO antibacterial effect [12,31], matrix degradation must progress sufficiently to make the nanotextured surfaces of the MgO particles fully available for contact with bacteria. This occurred really fast in the MgO/PLGA composites, within two hours, when bacteria were still in the lag phase [9,12] and before creation of biofilm has begun [32]. However, in the MgO/PLA the majority of holes were still too narrow for the MRs to get out even after 4 h of exposure to physiological medium. The uncoating of the MRs went even slower in MgO/PCL composites, where additional re-sealing of the formed holes could be observed (Figure 4h, Figure 8c), which can be explained by the low temperature of PCL glass transition (below −40 °C regardless of its molar mass [33]). The different rate of degradation was also expressed in the different kinetics and amount of magnesium release from MgO/polymer composites, which was the fastest and largest from the PLGA matrix (fastest degradation) and the slowest and lowest from the PCL matrix (slowest degradation) (Figure 3).

**(ii) Different distribution of MgO MRs inside the polymer matrix.** The analysis of the surface morphology revealed that the surface of the MgO/PLGA composite was more populated with MgO in comparison with the other two composites and MRs were more homogeneously distributed inside the PLGA matrix (Figure 1). As a consequence, once the thin outer layer of the polymer cover degrades, there are more exposed MgO particles and fewer sites without antibacterial protection in the more densely and homogeneously populated surface, such as in MgO/PLGA. Agglomerates (like in MgO/PCL, Figure 1d) contain a large local concentration of antimicrobial but leave many empty neighbouring sites with almost no antimicrobial particles. Thus, bacterial colonisation of these sites is not prevented. Moreover, larger accommodation of the outer polymer layer to the shape of the MgO MRs was noticed in case of MgO/PLGA, which was manifested as crumpling and wrapping around the particles (Figure 1b). Thus, the uncovered MgO MRs were more available for contact on the surface of the coating after the degradation of the outer thin layer in this case.

**(iii) Different extent of transformation to magnesium hydroxide.** This is probably the second most important contribution. Our previous investigation on antibacterial properties of different MgO materials has shown that the ability to resist hydrolytic transformation to hydroxide is key for improvement of antimicrobial properties as the hydroxide form is much less antibacterial than MgO [12]. This transformation occurs fast on the naked nanotextured MgO MRs in physiological solution, 50% of MgO gets converted within 2 h and the transformation is completed within 16 h [12]. However, this study has revealed that the MRs from MgO/PLGA (but not MgO/PLA or MgO/PCL) are already available for contact-based antibacterial action within 2 h of exposure to simulated physiological conditions, when they are still mainly in the MgO form (Figure 8a). Further, even though cracks due to degradation are not wide enough during the first 4 h in case of PLA and PCL to expose the whole nanotextured surfaces of the MgO MRs, they enable water to enter inside the composite. Thus, antibacterial nanotextured MgO MRs get transformed into less active Mg(OH)_2_ already inside the MgO/PLA and MgO/PCL composites, before an important fraction of particles is released and before they are available on the surface for contact-based antibacterial action (Figure 8b,c). By contrast, MgO MRs get released and/or exposed from the MgO/PLGA before they are inactivated by the hydrolytic transformation to hydroxide. Analysis of lethal kinetics of the MgO/PLGA composite has revealed that the major bactericidal action occurs already in the first 8 h (Figure 6a), during which the majority of MgO is not yet converted to less active hydroxide. In addition, it is also possible that the transformation to hydroxide is actually slower in the PLGA matrix, which might be connected with its faster degradability and consequent lower local pH around the MgO MRs.

**Figure 9:**
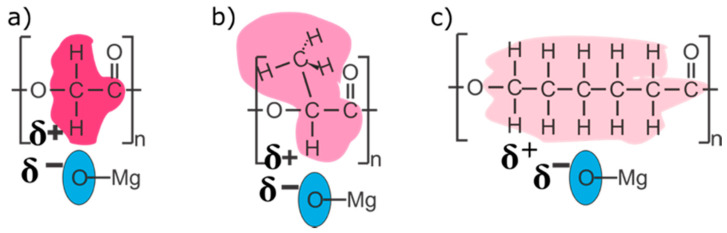
Interphase interactions inside the three different composites: (**a**) MgO/PLGA (only interactions with the PGA part shown), (**b**) MgO/PLA and (**c**) MgO/PCL.

**(iv) Different strength of interactions between the MgO MRs and the polymer matrix.** According to the IR analysis, the interphase interaction between MgO and PLGA predominantly involved interactions of C–H with the MgO surface (Figure 2). The strength of these interactions decreased in the following order: MgO/PLGA, MgO/PLA, MgO/PCL. They can be explained as a consequence of polar C=O and C–O bonds in the polyesters and redistribution of the positive charge from these two C atoms to the H atoms (and residual C atoms) by the electron inductive effect (Figure 9). Since the number of H atoms in monomeric units increases from 2 (PLGA), over 4 (PLA) to 11 (PCL), the partial positive charge on the H atoms is the largest in PLGA and the lowest in PCL (Figure 9). Inside the composite, the partially positive H atoms from polyester interact with the negative O^2−^ ions at the surface of MgO. The strength of this interaction is proportional to the partially positive charge of the hydrogen and directly affects the homogeneity of the distribution of the MgO MRs in the composite, but it also has other consequences. After the exposure of the MgO/PLGA to the surrounding medium, the upper layer was degraded while the interphase interactions between the MRs and the lower PLGA layer were still strong enough to retain them on the surface (Figure 4a and Figure 8a). Therefore, MgO MRs were exposed to the surrounding medium for the longest period of time before release (Figure 4), compared to the PLA and PCL. This effect is even more pronounced if we consider the transformation of MgO particles to hydroxide. After MgO is converted to Mg(OH)_2_, the interactions between the polymer and the MRs become weaker, so they escape from the MgO/PLGA composite. However, as the holes are not big enough yet in the PLA or PCL matrix, the hydroxide remains inside the coating, so it is detected by IR analysis. Further, when it comes out, it is retained on the surface for a shorter time than in PLGA, where MRs are still mainly MgO.

The fast bactericidal action of the MgO/PLGA composite coating relying on the fast degradation of the polymer matrix, together with the fast inactivation by transformation to hydroxide bring the concern about the prolonged antibacterial action of such coatings. The experiments performed on the aged MgO/PLGA coatings (inset in Figure 6a) have confirmed that the bactericidal action of the composite is decreasing with the increasing exposure of the coating to the physiological conditions. However, it still remained usefully strong after three days of aging. It is possible that the sustained action was provided by the homogeneity of the MgO MRs inside the composite, so that after removal of one layer by degradation another layer of nanotextured MgO MRs appears on the surface. It is not certain, whether the MRs deep inside (in the core of) the coating remain intact within the investigated period of time or water was able to reach them and every layer would come out with more hydroxide content, which would hinder prolonged action. IR spectra of the MgO/PLA composites exhibited disappearance of the hydroxide between 8h and 6 days of exposure to simulated physiological conditions even though the majority of magnesium has not been released yet from the coating, which suggests that the penetration of water into the coating is limited and the inner particles undergo hydrolysis only once they come closer to the surface layer. Further analyses on the sustained antibacterial action and the inner properties of the composites will be needed to confirm this.

The most important applicative value of this study is the confirmation (by a multi-method approach) of the strong antibacterial action of the optimal MgO/PLGA0.10 composite coating against a wide range of bacteria, including clinically relevant bacteria isolated from implant-associated infections, such as *S. epidermidis*, *S. aureus*, *P. aeruginosa*, *E. coli*, and multidrug resistant *S. aureus* (MRSA). The chosen strains are the most common species associated with the implantation-related infections [34] and the antibiotic resistance of bacteria is one of the most serious problems of humanity today [35]. The bacterial concentration used in the evaluation of the MgO/PLGA antibacterial potential was even two orders of magnitude higher than proposed by ISO 22196:2007(E), because the use of lower concentrations led to experiments with large standard deviations.

Moreover, MgO/PLGA0.10 composite was fully compatible with mammalian red blood cells, which is an important result regarding its cytotoxicity. Red blood cells constitute 99% of the blood cells [23] and are likely to be in contact with the surface of the implanted material [36]. In addition, we confirmed the ability of MgO to remove acidic degradation products of PLGA, in line with previous studies on the Mg-Al-Zn alloy/PLA composites [37]. After exposure to PLGA coating without MgO, the RBC membranes exhibited crenated shapes, called echinocytes, instead of normal biconcave discoid shape [38] (Figure 7b). The evolution of such shapes at the surface of the RBCs can be explained by the pH decrease, which is even more pronounced in the vicinity of the coating. Introduction of 10% of MgO within the PLGA matrix resulted in the reduction of the number of RBCs with crenated shapes and the pH remaining at 7.4. These results proved synergy of PLGA (carrier) and nanotextured MgO MRs (acid scavenger and antimicrobial) within the MgO/PLGA composite coating in achievement of clinically desired properties.

Nevertheless, at least cytocompatibility with other cells will have to be shown before this kind of composites can enter more applicative stages. Furthermore, evaluating the effect of adding nanotextured MgO MRs into the polymers on their mechanical properties and hydrophilicity will also be very important for biomedical applications. Recent investigations of MgO whisker/PLA composites have shown increased crystallinity, Young’s modulus, tensile strength and hydrophilicity of the composites in comparison with pure PLA [39]. The mechanical properties decreased during long-term degradation of the MgO whisker/PLA composites but their cytocompatibility was promising, with increased urine fibroblast (L929) cell viability on the coatings containing 2 wt% of MgO [40] and enhanced adhesion, increased viability and proliferation of osteoblast precursor (MC3T3-E1) cells on the coatings with 5% MgO content in comparison with pure PLA coatings [41]. Therefore, it would be wise to continue the investigation on the novel nanotextured MgO/polymer composites with further relevant tissue cell compatibility in vitro tests and in vivo tests. Especially the ways for elimination of the released MgO MRs and Mg(OH)_2_ from the body will have to be addressed.

## 5. Conclusions

A synergy between strong antibacterial activity against implant-related bacterial strains and mammalian-cell compatibility was achieved using an innovative design of 1D nanoengineered MgO structured in a biodegradable polymer matrix. The practical implication of this study is that the optimal design of biologically relevant MgO/polymer composite coatings requires the ability of the used polymer matrix to fully expose the MgO particles and retain them at the composite surface before their hydrolytic transformation to less active hydroxide occurs. This was obtained in the PLGA matrix, which provided the best combination of desired properties for the antibacterial protection. The MgO/PLGA composite containing 10% MgO exhibited strong bactericidal action against a wide variety of bacteria, including implantation-related clinical and antibiotic-resistant strains. From that standpoint it could be concluded that the proposed technology presents a promising approach for further applications and designing the surface of medical implants.

## Data Availability

Not applicable.

## Supplementary Materials

The following are available online at https://www.mdpi.com/article/10.3390/polym13132183/s1, Figure S1: IR spectra of the MgO/polymer composites after (a) 2-h and (b) 24-h exposure to physiological solution. Figure S2: ATR IR spectra of MgO/PLGA0.10 and MgO/PLA0.10 composites after 8 h and 6 days in 0.9% NaCl at 37 °C and linear shaking, Figure S3: (a) calibration curve for haemolysis determination. (b) RBCs on the PLGA and MgO/PLGA0.10 coatings as observed under optical microscope.
