# Supplementary material for: Antimicrobial Polymeric Composites with Embedded Nanotextured Magnesium Oxide
Source: Polymers (Basel). 2021 Jun 30;13(13):2183. doi: 10.3390/polym13132183 (PMC8271688; doi:10.3390/polym13132183)
Supplement: Supplementary file 1 [file polymers-13-02183-s001.zip › polymers-1235863-supplementary.pdf]

Antimicrobial Polymeric Composites with Embedded Nanotextured Magnesium Oxide

Supporting information

Nemanja Aničić,[1] Mario Kurtjak,[1] Samo Jeverica,[2] Danilo Suvorov,[1] Marija Vukomanović[1,*]

[1] Advanced Materials Department, Jozef Stefan Institute, Ljubljana, Slovenia
[2] National Laboratory of Health, Environment and Food, Maribor, Slovenia

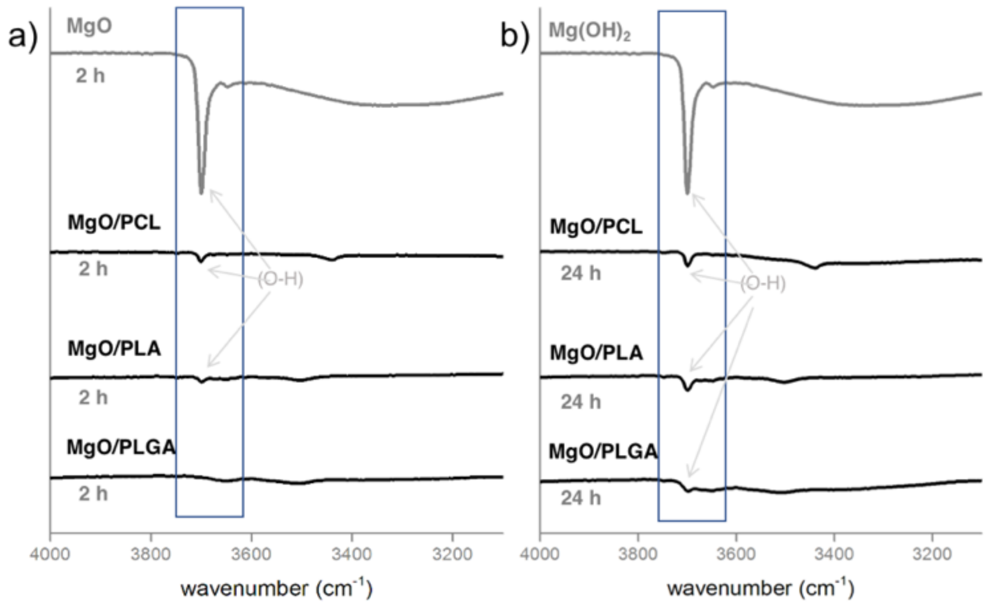

Figure S1. IR spectra of the MgO/polymer composites after a) 2-hour and b) 24-hour exposure to physiological solution.

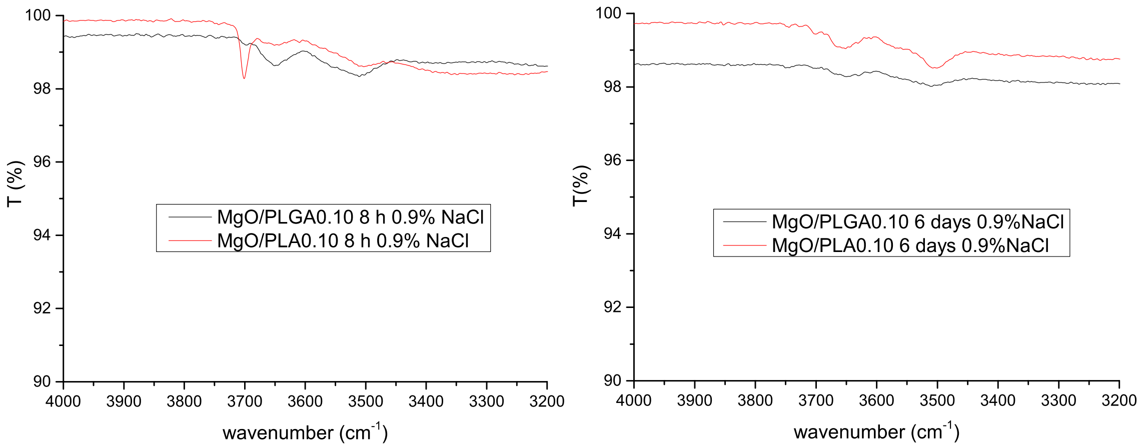

* Corresponding author: marija.vukomanovic@ijs.si , +386 1 477 3547

Figure S2: ATR IR spectra of MgO/PLGA0.10 and MgO/PLA0.10 composites after 8 h and 6 days in 0.9 % NaCl at 37 °C and linear shaking.

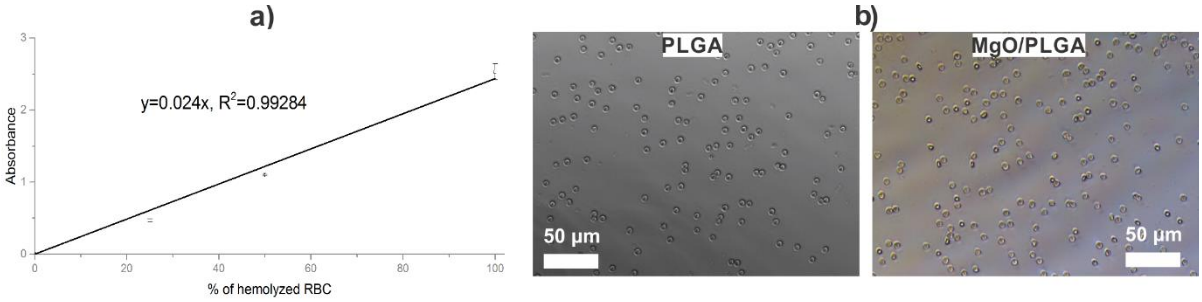

Figure S3: a) Calibration curve for haemolysis determination. b) RBCs on the PLGA and MgO/PLGA0.10 coatings as observed under optical microscope.
